# 

**Published:** 1895-10

**Authors:** 


### CONTENTS:

### SELECTIONS.

Article I.—The Scientific Spirit and The Ethics of Dental Practice. Dr. C. J. Essig - - - - - - 241

" II.—Treatment of Asiatic Cholera. Elmer Lee, A. M., M. D., Ph. G. - - - - - - - - 250

" III.—Plastics as a Bar to Bacteria. Joseph Head, M. D., D. D. S. - - - - - - - - - 258

" IV.—Duties of Dentist to Patient—Duties of Patient to Dentist. A. W. M'Candless, D. S. S. - - 263

" V.—A Practical Point in the Use of Disinfectants and Antiseptics. A. G. Young, M. D. - - - - 270

" VI.—How to Vulcanize Rubber Plates Between Metallic Surfaces. Dr. A. N. Dick. - - - - 275

" VII—Alveolar Abscess. - - - - - - 276

### COMMUNICATED.

National Association of Dental Examiners. - - - - - 277

### EDITORIAL, ETC.

Systemic Dissemination of Tuberculosis From a Patch of Lupus Vulgaris. - - - - - - - 281

Hypnotism in Surgery. - - - - - - - 282

Our Institutions. - - - - - - - - - 282

### MONTHLY SUMMARY.

Porcelain Faced Bicuspid Crowns. - - - - - - 283

The Importance of Prosthetic Dentistry. - - - - - 284

Fitting Crowns. - - - - - - - - - - 285

Beaded Enclosures in Vulcanite Dentures. - - - - - 285

Chronic Alveolar Abscess with Complications. - - - - 286

Pulp Capping. - - - - - - - - - - 286

For Setting Crowns. - - - - - - - - 286

Argonin. - - - - - - - - - - - 287

Pauperism. - - - - - - - - - - 287

A Vent in Your Port Polisher. - - - - - - - 287

Obtunding Sensitive Dentine. - - - - - - - 287

Alcoholism in France - - - - - - - - 288

Cyst of the Antrum or Highmore. - - - - - - 288

Inflammation. - - - - - - - - - - 288

